# Exploring the dimensions of responsible research systems and cultures: a scoping review

**DOI:** 10.1098/rsos.230624

**Published:** 2024-01-17

**Authors:** Sarahanne M. Field, Jackie Thompson, Sarah de Rijcke, Bart Penders, Marcus R. Munafò

**Affiliations:** ^1^ CWTS, Leiden University, Leiden, Zuid-Holand, The Netherlands; ^2^ University of Bristol, Bristol, UK; ^3^ Maastricht University, Maastricht, Limburg, The Netherlands; ^4^ Käte Hamburger Kolleg ‘Cultures of Research’ (CoRE), RWTH Aachen University, Aachen, Germany; ^5^ Department of Pedagogy, University of Groningen, Groningen, The Netherlands

**Keywords:** responsible research, research integrity, research practice

## Abstract

The responsible conduct of research is foundational to the production of valid and trustworthy research. Despite this, our grasp of what dimensions responsible conduct of research (RCR) might contain—and how it differs across disciplines (i.e. how it is conceptualized and operationalized)—is tenuous. Moreover, many initiatives related to developing and maintaining RCR are developed within disciplinary and institutional silos which naturally limits the benefits that RCR practice can have. To this end, we are working to develop a better understanding of how RCR is conceived and realized, both across disciplines and across institutions in Europe. The first step in doing this is to scope existing knowledge on the topic, of which this scoping review is a part. We searched several electronic databases for relevant published and grey literature. An initial sample of 715 articles was identified, with 75 articles included in the final sample for qualitative analysis. We find several dimensions of RCR that are underemphasized or are excluded from the well-established World Conferences on Research Integrity (WCRI) Singapore Statement on Research Integrity and explore facets of these dimensions that find special relevance in a range of research disciplines.

## 1. Introduction

Responsible conduct of research (RCR) is an aim that requires the synthesis of many disparate aspects. It draws upon, yet is distinct from, related concepts such as research integrity or responsible research and innovation (RRI). RCR casts a broader net than the main responsibility of research integrity, which is promotion of trust and confidence in research and its process. This broader net of RCR includes responsibilities that overlap with those of RRI, such as considering research's wider effects on society. However, while the concerns of RRI include the impacts on society of technological innovations and other research *outputs*, RCR focuses more tightly on the subset of responsibilities relating to the *process* of conducting research.

RCR is essential to the trustworthiness of research in general, and to the public's trust in scientific conclusions and aims. According to established responsible research frameworks (such as the World Conferences on Research Integrity (WCRI) Singapore Statement on Research Integrity [1]; henceforth referred to as the Singapore Statement^1^), the practice of RCR generally involves promoting rigorous research and confidence in the findings and fosters a positive research environment. Embedding RCR in research institutions and, more broadly, within disciplines, has great potential to boost the quality of future research, engender integrity in research practice and to enhance the culture of academic research.

Currently, however, we know little about what RCR looks like – or *should* look like—across disciplines. Currently, quantitative epistemic perspectives tend to dominate research discourse around integrity and responsibility. Take, for instance, the concept of reproducibility. It is a concept that arguably applies to many quantitative disciplines but is often perceived as less relevant or valid to some fields in the social sciences and humanities, particularly those which use qualitative approaches [2].

Concerns about mismatches between how RCR is conceived and implemented cast further light on the fractured understanding of how RCR ought to be defined, and to whom it applies. In their review on the research integrity and RCR practices literature, Aubert Bonn and Pinxten [3] show that most concerns are articulated at the level of systems, while most interventions and actions target individual researchers. This misalignment is recognized widely (e.g. [4]), and future endeavours to stimulate RCR practices should focus on the level of the research system. However, these structural levels are complex, and difficult to map and intervene at, while interventions that do target them are at risk of inadvertently still being operationalized at the individual level (for instance, as mentoring and teaching [5]).

Research in pursuit of diverse perspectives on what constitutes RCR across disciplines and cultures is maturing (e.g. [6]); however, the process is slow and needs further support. A crucial part of supporting the maturation process is recognizing that different epistemic communities assess the quality and moral status of research differently. Until the extent and character of these differences is better understood, the appropriateness and feasibility of proposals to reform research (e.g. open research, reproducibility) are unclear. This study aims to chart the existing scholarship on the diversity of RCR systems and cultures, which will inform further research on conceptualizing RCR and embedding those practices in institutional ecosystems. More concretely, we aim to highlight dimensions and principles of RCR that may feature in future frameworks on RCR. Our scoping review explores the main research question: what constitutes ‘responsible conduct of research’ in the literature.

### 1.1. Responsible conduct of research

We approach the subject of RCR from a background in science studies, metascience and research integrity, and we have published on the topic of epistemic diversity, responsibility and quality at length prior to this analysis (e.g. [2,5,7–9]). We are aware of the wealth of academic literature on RRI and have sought and continue to seek explicit inspiration there, as can also be read in this analysis. It is in this position that our decisions are rooted, from research questions to the choice of reference documents.

At this juncture it is necessary to also describe the role of this study as part of a larger project. Funded by Wellcome, our team is conducting a research project spanning 2 years to develop a diverse understanding of RCR as it is conceptualized and applied across and between different epistemic traditions and disciplines in research. Building on this scoping review, we will next endeavour to create a mapping of this understanding of RCR through a Delphi study with a panel of RCR experts from a broad range of disciplines across the research sphere. Following this, we will focus on piloting ways for enacting this understanding as practice, by embedding it in RCR communities of practice across the UK and part of Europe. The mapping developed in the first half of the project provides a scaffold for the activities of the communities of practice in the latter half. Importantly, it can be used more broadly to assist other interested entities (such as individuals or research groups) in conceptualizing and applying RCR principles in their own research and pedagogical contexts. We aim to cast our epistemic net broadly in the development of the RCR map, hoping to generate a tool which is helpful for a broad perspective on the research sphere while at the same time being specific enough for practitioners, such as researchers, to adapt to their own practice.

## 2. Method

In line with the motivations for conducting scoping reviews set out by Mays *et al.* [10] and Arksey and O'Malley [11], we conducted this scoping review to rapidly map out the key concepts of our research topic and identify possible gaps in existing scholarship, using the main sources and types of evidence available. Specifically, we undertook a scoping review of the concept of RCR and explored how it is embedded in different disciplinary practices in science, with existing RCR frameworks as a reference point, or ‘springboard’.

Arksey and O'Malley's [11] framework recommends undertaking the scoping review process in six stages: 1. Specify the research question, 2. Identify relevant literature, 3. Select studies, 4. Map out the data, 5. Summarize, synthesize, and report the results, and 6. Include expert consultation. We applied the first five steps, but we did not seek out expert consultation. The Delphi study following on from this one that we mentioned in the introduction section involves expert consultation that we will draw on in subsequent stages of the overall project. We largely adhere to the Preferred Reporting Items for Systematic Reviews and Meta-Analysis—Extensions for Scoping Reviews (PRISMA-ScR) for reporting guidelines [12].^2^ The protocol for the scoping review can be found on the Open Science Framework: https://osf.io/vy684.

### 2.1. Search strategy—identifying relevant literature

Four scientific literature databases were searched: Web of Science (WoS, core collection), PsycINFO, Social Science Research Network (SSRN), and the Wiley Online Library (WOL) using keywords (primarily ‘responsible research’ and adjacent concepts like ‘research quality’ and ‘research integrity’). The databases were searched between 15 and 20 September 2022, and the search included all articles up until the date of the search itself.^3^

These databases contain a wide variety of literature from many disciplines, including ones that emphasize humanities and the social sciences. We searched the Sage Research Methods Online (SRMO) as well; however, the database yielded, unexpectedly, no results for the search strings in the title or abstract fields. Casting a wide epistemic net was crucial to the aims of this study, and we chose these databases with that in mind. Additionally, we selected databases that we had full access to through our institutes' library subscriptions. While the JBI Scoping Review Methodology Group recommends at least two databases for a scoping review, we chose four databases to ensure a comprehensive coverage of the literature.

We searched the grey literature using the same keywords as in the systematic searches described above. Large university dissertation databases, focusing on EU and UK-based institutes (such as the University of Amsterdam Scriptie database), as well as other databases for grey literature such as ProQuest, EBSCO Open Dissertations and the Grijze Literatuur in Nederland (GLIN) databases were searched.

Additionally, we used snowballing search strategies to identify other relevant literature that the systematic searches might have missed. We searched included articles’ reference lists, and explicitly search for the work of key authors (such as Aubert Bonn) and organizations (such as the Netherlands Research Integrity Network; NRIN) to add to our database. We consulted the Peer Review of Electronic Search Strategies (PRESS) checklist [13] to help ensure that our search strategy would yield a valid body of literature.

### 2.2. Study selection

We screened texts discussing the RCR framework or practices that were backed by some degree of consensus; that is, all sources that were peer-reviewed, part of official policy documentation, or certified in some way (such as the grading of a PhD dissertation) were considered eligible. We included original research that involved primary data collection (qualitative or quantitative) and secondary data analysis (on an existing dataset), as well as reviews, editorials and letters to the editor. We included perspectives, narrative and historical pieces (though the search yielded no articles of this nature). We also included doctoral dissertations, policy documents and other relevant documentation such as organizational codes of conduct (which tend to reify the values and norms of groups).

We explicitly excluded texts such as preprints, pre-registration documents, study protocols and blog posts which have not been endorsed or reviewed formally^4^ in any fashion, or that have not been submitted for publication. This restriction was in place to ensure that only documents that have at least a rudimentary endorsement within the scientific community are included in the database. The grey literature was only eligible for inclusion if it was published in some form by a recognized group or institution and available in a formal repository (for instance, on a university or other institution's website). We also excluded conference abstracts and posters that were not accompanied by a full text. As our search covered the titles and abstracts of texts, those without either of these elements were excluded.

It is worth noting that RCR is closely linked to the Responsible Research and Innovation framework (RRI). Although we do not intend to detangle RCR from RRI (cf. [14] for why this is unlikely to succeed), and included articles that discussed the whole RRI framework, we did attempt to exclude articles whose full texts clearly focus only on the I (i.e. innovation) aspect of RRI.

The remit for our search was relatively inclusive, though we were limited to sources that were written in English, Dutch and German (as these are the languages that our team collectively has sufficient command of). We largely limited our search to material we could access online. We did not have the time to easily obtain all print-only copies of all potentially relevant resources; however, we did not come across any relevant texts that were only available in print; everything was available online.

### 2.3. Charting the data

Following the first two searches, the details of all articles identified were uploaded into Zotero (desktop client, version 6.0.19) and were checked to ensure that the search results were appropriate for our purposes. This check clearly indicated that we were finding the kinds of articles we were interested in. During this audit, all duplicates were removed. Two of us (J.T. and S.M.F.) audited the records. We each checked half of the total sample to check that all inclusion and exclusion criteria were met during the abstract and title screening. S.M.F. and J.T. discussed disagreements and uncertainty about the screening and resolved the discrepancies without the need for a third opinion.

**Figure 1.**
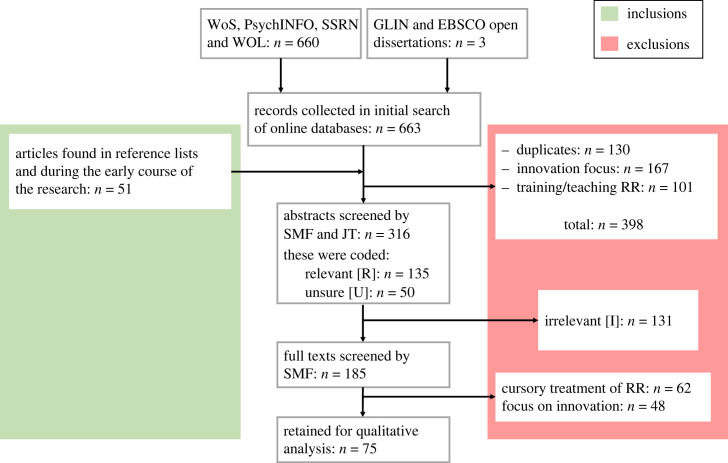
PRISMA flowchart depicting inclusions and exclusions across the data collection and screening phases in the sample of articles. Red shading highlights exclusions, while green highlights included articles.

All full texts of sources deemed suitable for the scoping review were retrieved. These records were then checked against the inclusion and exclusion criteria in a process similar to that of the aforementioned audit by S.M.F. Reasons for exclusion of full-text resources were documented in a log. The process of the search and inclusion/exclusion stages is explained in the text and depicted using a PRISMA-style flow diagram (figure 1). We created a form to capture all data of the extraction process in Microsoft Excel.

The form contains article titles, year of publication, authors, publishing outlet and abstract. We had planned to include conceptualizations of RCR in the articles; however for this literature it proved too difficult. Often, authors did not clearly state their own conceptualizations, or there were several conceptualizations that were interconnected. The idea of extracting such information is feasible for some literature corpora that contain mostly empirical studies or clearly defined definitions; however, we found that it was less feasible for our own case.

## 3. Corpus

### 3.1. Data collection

As the PRISMA flowchart in figure 1 shows, a total of 714 articles were obtained. A total of 661 were from the WoS, PsychINFO, SSRN and WOL (508, 92, 41 and 20 articles were returned, respectively). One of those from WoS was discarded immediately as it was in Hungarian. Three were found in the grey literature databases: GLIN and EBSCO Open Dissertations. A further 39 were found through searching the reference lists of existing records in the sample during the full-text screening process. Finally, 12 more articles were added to the database as they were identified by the authors incidentally during the period of searching and screening (through other research being done on the topic at the same time). Of these, 130 were duplicates and therefore excluded. Of the remaining 584, a further 268 were excluded as they were clearly heavily focused on the innovation aspect of RRI (which was outside of the scope of our review) or focused on issues surrounding the implementation or training or teaching of RCR rather than defining the concept or exploring elements of RCR in terms of its definition or concepts.^5^ This leaves a total of 316 articles which made it into the abstract screening process. This process was undertaken by S.M.F. and J.T., who used the ‘tag’ function in Zotero to mark which article abstracts indicated a relevant article [R], an irrelevant article [I], or that the screener was unsure whether the article was relevant or not [U].

Of the 316 article abstracts screened, 135 were marked as relevant, 131 irrelevant and 50 unsure. The full texts of the articles coded as relevant and unsure were imported into ATLAS.ti (version 22) by S.M.F. for full article screening, and qualitative coding and thematic analysis of the texts of articles included in the final sample.

During full text screening, which was conducted solely by S.M.F., 110 of the 185 articles screened were excluded. As mentioned before, several were excluded because they focused too heavily on the innovation aspect of RRI to be relevant for our analysis. Others were excluded because although they focused on RCR, they were unlikely to advance our research questions due to their content: this included articles which only mentioned RCR cursorily, discussed it on a very superficial level, or mentioned it indirectly. A small number of articles were excluded because they were duplicates in terms of large chunks of their text, despite their abstracts and titles, and sometimes even author lists or orders being different. Some of the more prominent RCR researchers appear to re-use large sections of texts across different (especially policy) documents, which seemed to be behind this phenomenon.^6^

### 3.2. Descriptive information

At the end of the selection process, 75 articles remained for qualitative coding. See appendix A for a full reference list of these articles. These articles were mostly essays and perspective or opinion pieces. Several articles featured suggestions for codes of conduct or ethical guidelines, and some contained case studies from which authors drew ‘lessons' or used the case studies to highlight important considerations for RCR. A few were policy documents or accompanied or supported policies. Many grappled with emerging challenges in responsibility for new or developing fields and some presented new dimensions of RCR or featured empirical research studies.

The articles spanned several decades, with the oldest article being from 1993 and the most recent from 2022. The distribution of these shows an increase in discussion about RCR in the literature (at least across the databases we searched) over the range of years in which the articles were published. The median year of publication for both the full sample and the final sample of 75 articles is 2017, and both means are 2015. This is evidence of a left-skewed distribution and shows that the sample is heavily populated with more recent articles, with half having been published in the last 6 years of the range (30 years for the full sample, and 29 for the final sample). The range and distribution are consistent with the overall range of the sample that was processed during full screening before exclusions, which indicates that our exclusions removed articles in a likely unbiased fashion across the sample.

## 4. Analysis

### 4.1. Approach

S.M.F. conducted a thematic analysis using the content of the 75 articles in the sample, guided by the methodology of Braun and Clarke (e.g. [15,16]). Using ATLAS.ti (version 22 for Mac OS; 2022), codes were applied to salient elements in each text, then were grouped together to create themes. In this case, ‘salient’ referred to parts in article texts that implicitly or explicitly defined or explained RCR.

**Figure 2.**
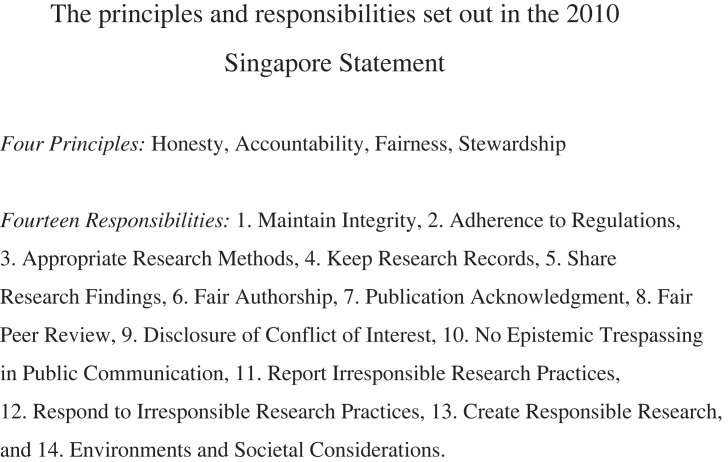
Textbox containing the four principles and 14 responsibilities of the Singapore Statement (simplified and paraphrased by B.P.).

This analysis was guided by ‘sensitized concepts' [17] which featured in the RCR literature that S.M.F. was already familiar with (primarily the Singapore and Montréal Statements). These sensitized concepts laid the foundation for the analysis of the data that were collected. Although some of the themes we discuss corresponded to RCR dimensions already identified in the Singapore Statement (figure 2 presents the principles and responsibilities featured in it), most of them arose directly from salient dimensions in the articles' content. In this way, the coding process is a hybrid of the inductive and deductive approaches. S.M.F. chose this approach so that both the Singapore Statement and the literature could inform a new set of dimensions, without being constrained by a pre-specified coding strategy. The themes were generated semantically (based on the ‘face value’ of the words in the text) rather than through interpreting possible latent patterns in the data.

This approach links back to our overarching aim, which was to map the current literature on RCR across disciplines in the context of the broader Wellcome project's plan to develop and apply RCR concepts across research disciplines. We wished to gain a sense of what aspects of RCR are cross- or inter-disciplinary, and which might be discipline-specific. We also wanted to get a sense of what might be missing from existing RCR frameworks such as the Singapore Statement, and what meaningful departures from the Singapore Statement might be worth exploring in future RCR framework development.

### 4.2. A note on existing frameworks

The first step in understanding what RCR is, using the literature collected in this review, was to explore how the articles engaged with topics covered in the Singapore Statement. Our project uses this well-established 2010 framework as a starting point from which to broaden and update our understanding of RCR. The Singapore Statement has undoubtedly had an impact on RCR since its establishment (for instance, in his 2015 report on the impact of the Singapore Statement, Axelsen credits it with helping Denmark handle its first major known case of research misconduct, calling it a ‘concise, short and authoritative’ guide for action).

That said, it is, to some extent, difficult to embed in research communities in practice (our goal, previously stated, is to establish a knowledge base for this purpose). This is partly because the Singapore Statement is relatively general and even vague in parts, and some of the responsibilities effectively amalgamate several distinct elements of RCR together. This latter issue makes it harder to action in practice for targeted behaviour and perspective change (the development of a targeted framework is one goal of this project). That the Singapore Statement is broad and vague is not surprising; it was designed to be widely used as a general guide to policy and conduct, and largely focusing on aspects of research integrity (as its full name suggests), which means that it cannot be expected to encompass *all* aspects of RCR.

It should also be noted that the Singapore Statement is a product of the scientific landscape of more than a decade ago. As a result, it reflects the values and priorities of a research culture that existed before movements focusing on open science and science reform began to gain their current momentum. This implies that it may not address the priorities of newer research areas, and fields that are at the forefront of modern human innovation such as nanotechnology, stem cell research and artificial intelligence. The same might apply to a heightened contemporary focus on diversity, inclusion and respect of indigenous lands and peoples. Furthermore, the 14 responsibilities set out in the Singapore Statement (figure 2) relate largely to the actions of the individual researcher, despite a growing recognition of the responsibilities of institutes and governing bodies and the cultures they engender in research practice integrity and scientific output quality.

We also emphasize that while the Singapore Statement is widely used, it is not the only framework to set out responsibilities for researchers and institutions. For instance, the WCRI has released three other frameworks relating to RCR: the Hong Kong Principles, the Amsterdam Agenda and the Montréal Statement.^7^ Rather than providing guidance to improve research practices, as the Singapore Statement does, the Hong Kong Principles are aimed at helping institutions to minimize perverse incentives and other practices that foster questionable research practice. The Amsterdam Agenda is all about evaluating RCR practices—it sets out a method of assessing efforts to improve research integrity and the use of empirical information in developing research integrity policies. Finally, the Montréal Statement focuses on practices to foster research integrity in contexts that specifically involve cross-boundary collaborations. Though none of these other frameworks are as useful for our purposes as the Singapore Statement, the Montréal Statement does reinforce some dimensions of RCR relating to research with indigenous peoples and objects and was used to help inform the coding strategy we discuss later.

The European Code of Conduct for Research Integrity (the current version is from 2023), published by the All European Academies ([18] working group on science and ethics and the European Science Foundation, is another example of a well-known framework for RCR practice. This framework is recognized by the European Commission as the reference document for integrity in research practice for all EU-funded research and refers specifically to research in European institutes and by individuals working within them. It aligns with the Singapore Statement in its content and structure, and heavily relies on that and the other WCRI frameworks discussed above. It features four principles (reliability, honesty, respect and accountability) and several ‘good research practices' relating to eight different research contexts (the research environment; training, supervision and mentoring; research procedures; safeguards; data practices and management; collaborative working; publication and dissemination; reviewing, evaluating and editing). It also discusses violations of research integrity.

To that end, we will use the Singapore Statement as a scaffold for our scoping review, noting where we find themes that accord with or refine the responsibilities set out in it, as well as adding more concrete and specific elements of RCR practice which have come to light from the literature of disciplines outside of the behavioural sciences. We discuss dimensions that might be treated as core principles in subsequent frameworks on RCR, and others that may function better as concrete individual responsibilities. This may be useful for individuals and collective entities with an interest in applying RCR.

### 4.3. Dominant themes

One consistent finding across the articles is that they tend to highlight that the elements of RCR considered by the Singapore Statement are, in practice, highly complex and multifaceted—as well as being malleable and discipline-dependent. Another finding is that the Singapore Statement does not explicitly recognize some concepts that the literature body in our sample considers central to RCR. This is probably associated with the discussion above about the generality of the Singapore Statement, as well as its focus being on research integrity rather than exploring all elements of research conduct. It is also evident that different fields use different terms to describe common concepts.

Below we will discuss six dominant themes in the analysis: integrity, accountability, reflexivity, transparency, anticipation and capacity-building (extra information on themes and code groups can be found in appendix B). We will subsequently reflect on how including these themes in RCR frameworks can ensure these frameworks' relevance to researchers and institutes across a greater number of disciplines, and update RCR practice guidelines.

### 4.4. Integrity

The theme of integrity surfaced repeatedly in the literature we surveyed, emerging as a wide-ranging concept that is key to the very idea of RCR. At least 20 articles in our corpus discuss integrity in this vein. Most of these, unsurprisingly, come from the corpus of literature on research integrity. Some sources explicitly position integrity as central to RCR; for instance, Horn [19] describes integrity as a value upon which science is built. Antes and Maggi [20] point out that research integrity is often used interchangeably with the concepts of RCR and research ethics in the literature.

Other sources speak more implicitly to the importance of integrity in RCR, by describing integrity as a multifaceted concept or using it as an overarching term covering many other aspects of RCR. Valkenburg *et al*. [5] directly refer to integrity as such, defining it as ‘a collection of qualities that researchers and research institutions must possess, to ensure that research produces valid and reliable scientific knowledge, in a way that is societally desirable, and with a proper positioning of scientists in society’ (p. 2). They also discuss ‘sanctionable values' (that is, values that can be enforced, and which wreak undesirable consequences if not prioritized), such as human dignity, fair credit, transparency and the avoidance of falsification, fabrication and plagiarism. Similarly, Nijhuis and de Vries [21]), considering RCR in the context of landscape architecture, suggest that integrity includes multiple components: ‘… the design process must meet five criteria to ensure integrity and validity: purposefulness, reliability, consistency, transparency, and usability…’. (p. 100). Discussing conduct in collaborative vaccine studies, Kurz *et al*. [22] indicate that integrity is built upon other underlying elements: ‘scientific integrity means acting in accordance with the values of science, such as truthfulness, honesty and open reporting’. Loomis and colleagues [23] cite this same definition of scientific integrity in their paper on RCR in marine science (electronic supplementary material).

The literature as a whole not only stretches the definition of integrity to accommodate a large number of subsidiary concepts; it also varies in how it conceptualizes what integrity is at its core. In addition to defining integrity as a collection of qualities, as mentioned above, various sources conceptualize integrity either as a set of values, as an intention or motivation, as actions, or as a habit. Situating integrity as a value or set of values is perhaps the most common. Horn [19] defines integrity as a value upon which science is founded, while Kurz *et al*. [22] define integrity as an alignment with the values of science. More subtly, Antes and Maggi [20] suggest that integrity is often conceived of as part of an individual's personal values, whereas Valkenburg *et al*. [5] link integrity to the ‘sanctionable values' discussed above. Moving from values to internal states, participants in an empirical study by Haven *et al*. [24] described integrity as ‘having the right type of intrinsic motivation: the desire to do good research and to pursue the truth' (p. 3024). Antes and Maggi [20] similarly suggest that integrity is related to ‘having the right intentions’. Other sources frame integrity as rooted in behaviour; for example, Kurz and colleagues [22] describe integrity as action, specifically ‘acting in accordance with the values of science', while Horn [19] posits that integrity implies a habit of honesty.

Most of the articles in our corpus, including those discussed so far, situate integrity as a part of the conduct or process of RCR; however, some conceive of integrity instead as an outcome or goal of RCR. In a position paper on a vision for responsible research in business management, the Community for Responsible Research in Business and Management states that acting with responsibility ‘protects the integrity of science’ [25, p. 7]. This echoes Wager and Kleinert, who write that one responsibility of research editors is to safeguard the integrity of the scientific record [26]. Both present integrity as a desirable outcome that RCR can facilitate. Nijhuis and de Vries [21] embrace a similar framing, albeit more subtly: they state that a responsible design process must include several criteria in order to ensure integrity. Finally, De Peuter and Conix [27] propose a culture of integrity as an ideal that can be achieved by transforming scientific conduct through drivers such as RCR education and training.

Across the literature, integrity is construed as operating at various levels, from the individual to institutional to cultural and societal. Individual framing is common; for instance, Horn [19] describes integrity as ‘an essential quality of a good researcher’ (p. 22), whereas Antes and Maggi [20] link integrity to personal values and intentions, as well as presenting integrity of the research itself as a responsibility of authors. Similarly, Wager and Kleinert [26] frame integrity as something that must be guarded by research editors. Returning once again to Valkenburg *et al*. [5], it is evident that to them integrity is not only an individual responsibility, but one that equally applies to institutions and involves a wider relationship with society. They argue that culture and practice play a role in the relationship between these two levels of individual and institutional responsibility for integrity. Similarly, De Peuter and Conix [27] explore integrity as a product of research culture, implicating institutions as responsible for fostering integrity, and describing how the scientific community plays a role in shaping the construal of integrity as more than simply punishing misconduct.

Overall, the literature indicated that researchers from many disciplines consider research integrity a foundational part of RCR that spans many elements. However, this treatment sits somewhat at odds with narrower definitions of integrity in existing frameworks, such as the Singapore Statement. Although the Singapore Statement is framed, in its whole, as a statement on research integrity, it separates out integrity as a single responsibility among 14 and provides a relatively simple definition of it, linking it to trustworthiness (researchers should take responsibility for the trustworthiness of their research). This contrast with the much wider treatment of integrity in the literature raises the question: which scope is more appropriate for future RCR frameworks?

The answer may lie somewhere in between. Although the literature clearly shows that integrity encompasses more than just trustworthiness, its treatment as an umbrella concept welcoming various responsibilities, virtues, structures and responsible practices has rendered it so diffuse a concept that it risks becoming meaningless. In other words, integrity may have limited applicability as a practical dimension of RCR on its own, without being explicitly linked to practical behaviours and attitudes that may be recommended for researchers and institutions to adopt.

### 4.5. Accountability

Accountability has to do with accepting responsibility for one's actions, including, in this context, one's research practice and more generally, behaviour in the research sphere. In line with this, Pellizzoni [28] writes that accountability is about ‘building relations of answerability and justification’ around research conduct. Accountability is one of the Singapore Statement's four principles and, just like integrity, it is a concept that is threaded through several of the Singapore Statement's responsibilities. For instance, responsibility 6 suggests that researchers should take responsibility for their contributions in published work, and responsibility 14 discusses the ethical obligation researchers have to society, to balance the risks and benefits of scientific advancement.

Despite this, accountability is not clearly defined in the Singapore Statement. This makes it seem like an intractable concept; difficult to apply in practice. This is, at least in part, because RCR is relational. Researchers are never sufficiently clear about what they are accountable for, with whom they share that responsibility, to whom they are accountable and why, and how the account they give should look. Unsurprisingly, it is a prominent theme in RCR practice for several disciplines, including research with indigenous populations, environmental research and nanotechnology,^8^ as these areas have greater potential for research (and researchers) to cause harm. In contexts like these, and others, many articles clearly define accountability as a tractable dimension of RCR and provide examples of approaches to embedding accountability in research practices. Across various sources, accountability is treated as a vital element of aims both to ensure trustworthy research, and to align research with the good of those involved, and wider society.

Swaen and colleagues [29], who lay out guidelines for responsible epidemiologic research practice, provide some boundary conditions for accountability. They point out that accountability is part of the entire research process, from start to finish, and not only restricted to the period of collecting data. They write that although the study's corresponding author is charged with answering questions about the study, accountability encompasses the contributions of all research group members (in line with responsibilities 6 and 7 of the Singapore Statement) and is not restricted to individual contributions. In other words, all authors are responsible for the whole work, rather than only the part they worked on. We need to acknowledge here how challenging such an endeavour can be, especially in some large team science projects where some authors do not have the knowledge or skills to verify the work of other authors. Swaen and colleagues link accountability with transparency, declaring that future accountability is enabled by being transparent about the research process as it has unfolded.

Accountability is highly relevant for those who conduct research with indigenous populations and on indigenous or sacred sites, and those who conduct participatory research. This is partly due to existing tensions between indigenous groups and research groups (caused by previous harm through irresponsible practice on the part of researchers and institutions), and partly because the co-production of knowledge yielded by research with indigenous peoples and lands is only achieved through the accommodation of different, and sometimes conflicting, worldviews.

Matson and colleagues [30], who conducted participatory research with first nations communities in the Great Lakes region of North America on the topic of *Manoomin* (a breed of wild rice that is a sacred food to many tribal groups and which faces decline due to multiple environmental stressors), have much to say on the matter of accountability and research with indigenous groups. From this participatory relationship they derive 10 principles of responsible research, throughout which accountability forms a strong theme. They emphasize the need for researchers to be held accountable to communities they are conducting research with and in, which is crucial to ‘… enable robust, nuanced, and effective environmental science, policy, and stewardship’ (p. 109). They also warn that while concrete documents such as memoranda-of-understandings and protocols are important for accountability, as they set out agreements and expectations for conduct, they do not supersede the development and maintenance of relationships between indigenous communities and researchers—participation which requires regular communication, sharing, respect and trust.

Accountability is also important for those who work with the environment and in nanotechnology. Loomis *et al*. [23] argue the importance of a code of conduct for certain research in the marine sciences. In the supplemental material associated with their article, they highlight the need for accountability in this context, using a quotation from the European Commission [31]: ‘Researchers and research organizations should remain accountable for the social, environmental, and human health impacts that their [nanosciences and nanotechnologies] research may impose on present and future generations’ (p. 7).

The literature also treats accountability as relating to processes and stakeholders beyond the context of individual researchers conducting their research. In a discussion of accountability within the research publishing process, Wager and Kleinert [26] emphasize that accountability for authors, reviewers and editors is achieved in part by closely following appropriate policies, standards and requirements, which presumably set out guidelines for other practices that enable accountability. They argue that authors must be held accountable both for the integrity of the research study, and for the reporting of the study. Aubert Bonn *et al*. [32] show that although it is not as salient a focus for institutions as, for instance, openness and excellence, accountability is often explicitly referred to as a guiding principle by a sample of 18 leading research universities in Europe.

### 4.6. Reflexivity

Reflexivity's meaning varies depending on how it is applied. In many cases, when referring to the method of a qualitative researcher, it refers to the process of reflecting on oneself, especially in one's role as a researcher in the research process [7]. This individualistic, somewhat ‘introverted’ [33] meaning is still applicable when it comes to RCR dimensions that relate to individual research practice; however, Wynne provides another definition that is more easily applied in the current context. He writes that reflexivity can be the ‘process of identifying, and critically examining (and thus rendering open to change), the basic, preanalytic assumptions that frame knowledge-commitments’ [33, p. 324].

Reflexivity is not part of the Singapore Statement, even implicitly. This is not surprising, as it is only relatively recently that reflexivity has come into the spotlight, from being an obscure qualitative research method to being a part of reforming scientific practice more broadly (including in quantitative research)—an assertion borne out by the findings we discuss shortly. It is still missing from many formal RCR frameworks, despite increasing acknowledgement in the literature of its importance. McLeod [34] points out that ‘[…] little space is allowed for reflection on personal values' (p. 11) in research; however, she emphasizes that it should take a prominent place in RR(I) frameworks, as an ‘. . . obligation for researchers to reflect on the values that underlie their own work and broader governance system’ (p. 10). Similarly, Valkenburg *et al*. [5], writing on research integrity, explain that reflexivity is ‘… needed to be able to deal with the moral complexities that research work inevitably comes with’ (p. 14). They also highlight that though reflexivity is certainly an individual and institutional responsibility, it should be included in RCR practice.

Many of the sources mentioning reflexivity came from the RRI literature. In a discussion on privacy and RCR, Stahl [35] notes that RR(I) must be reflective if its development and implementation are to be successful. This is because reflexivity can help prevent errors, highlight gaps and distortions, and provides early warnings for problems which can be corrected. Its assumptions should be made explicit, contends Stahl. Forsberg *et al.* [36] share similar sentiments on the importance of reflexivity, positing that ‘reflection on underlying values' is a central part of RR(I).

Sources we surveyed also see reflexivity as instrumental in one of the main aims of RRI, to align research with wider society. Wynne [33] argues that reflexivity (on the part of institutions in particular) is crucial for improving relations between science and society. In his analysis, he finds that science ‘… frequently incorporates and is shaped by implicit models of user-situations or social practices’ (p. 334); however, these models do not always reflect reality, and that mismatch in turn harms the ‘validity and public legitimacy’ of research, alienating society from science. Worse, these incorrect implicit assumptions underlying research can turn prescriptive as knowledge is turned into practice, compounding the problem. He turns to reflexivity to bridge this disconnect, writing that reflexive institutions could help circumvent the alienation of society by unquestioned scientific assumptions and the problems it causes.

In a much newer article, Gwizdała and Śledzik [37] also explicitly link reflexivity (both individual and institutional) with science's interactions with the public. They discuss an association between reflexivity and ‘public dialogue, science and public collaboration’, stating that reflexivity turns responsibility into a ‘public matter’ (p. 62). This is because reflexivity requires that individual researchers and organizations reflect on their own assumptions (political, social and ethical), which prompts consideration of their own roles and responsibilities, especially when it comes to dialogue with the public, and framing problems and solutions that affect them. ‘Promise management’, discussed by Zwart *et al.* [38] in their article on the origins of and changes wrought by the RRI framework to European research governance, interacts closely with reflexivity (see also Penders & Goven [9], who refer to the ‘stewardship of scientific promises'). Managing the promises made to—and expectations of—society is important (however, due to chronic overpromising in the past, science and technology are facing a ‘credibility deficit’, contend Zwart *et al.* [38]). Reflexivity may help researchers and institutions keep better track of what promises have been made, to whom, and whether or not they have been met. It can help the framing of promises so that they are more plausible, reliable and robust, and can help ensure that these promises ‘concord with the needs and expectations of society’ [38, p. 18].

Reflexivity was also seen as instrumental to other RCR practices, such as anticipation. Fisher and Mahajan [39], considering responsibility in the context of nanotechnology research, suggest that reflexivity can help deal with uncertainty surrounding research implications. They discuss that both the applications and consequences of research are unclear in the context of socio-technical integration, and that reflexivity can be used during the research process to help anticipate downstream effects of innovations as researchers take the time to actively think about them. Gwizdała and Śledzik [37] link reflexivity and anticipation in a similar fashion, also in the context of technological development. They write that exercising both anticipation (see below) and reflexivity together helps circumvent the risk of making incorrect predictions in the early stages of technological development.

### 4.7. Transparency and openness

Transparency (a word interchanged with ‘openness’) is another dimension of RCR that is only once and briefly referenced in the Singapore Statement (we refer to responsibility 5) yet features heavily in the literature across multiple disciplines, mainly regarding increasing trustworthiness and validity of research. This may reflect increasing general scientific interest in openness due to the open research and scholarship movement [40] over the past decade. Tijdink *et al.* [41] call open science a ‘trend’ in the research community, and they argue that examining its advantages and disadvantages should be a priority for future research. Generally, transparency in the context of research practice refers to making the research process (including methodology and analysis strategies, for instance) and research products (including books, articles, data, and analysis code for instance), as openly accessible as possible (or at least, where openness is not possible or may threaten the privacy of participants and infringe upon research ethics agreements, adhering to the FAIR principles—meaning to make digital assets findable, accessible, interoperable and re-usable^9^). Many articles in the corpus featured in this scoping review consider transparency, or openness, to be a key research responsibility. Several of them (e.g. [20,21,42]) discuss transparency as being a meta-responsibility, in that acting with transparency when conducting, reporting, governing and assessing research helps one implement other responsibilities such as honesty, accountability, trust and ethics (which will be discussed shortly). Indeed, transparency would function in an updated framework very much like one of the Singapore Statement's four research integrity principles; that is, like integrity or accountability, it is more of a core principle that affects practice in several more specific and concrete ‘responsibilities’ rather than being a responsibility in and of itself. This is reinforced by Haven *et al.* [24], whose empirical article shows that researchers believe openness is a vital part of an ideal research climate and ought to be implemented as part of practice in many different researcher behaviours.

While openness and transparency can be viewed as the simple practice of sharing one's research materials, data and outputs, their significance as meta-responsibilities are complexly tied to different challenges in different research disciplines. Assen *et al.* [43], who explore researchers' and patients’ conceptions of RCR in the context of stem cell research, report that transparency is seen as an important aspect of dissemination which can help manage expectations and ‘hype’. Mittelstadt [44] discusses applying RCR principles to healthcare technologies, where difficult issues of privacy and ethics must be navigated with responsibility. He writes that transparency facilitates users' assertion of their rights to privacy, including control over their own data, and decision-making about acceptable use of those data (though, paradoxically, this can lead to users’ decisions to *not* share their data, thus limiting openness!).

It is worth noting that openness does not always refer to transparency in the context of RCR. For instance, in their article on *Manoomin*, Matson and colleagues [30] discuss openness as the state of being or becoming open to the worldviews of others. Naturally, for much research, this is a relevant interpretation of the concept, and certainly for any research that involves engagement with indigenous or protected populations, or unique communities of individuals. This element links to reflexivity, the practice of which can lead to recognizing the impact of underlying values, which differ in other communities and cultures.

### 4.8. Anticipation and harm minimization

Anticipation is another aspect of RCR that, while pervasive in the RRI literature featured in this review, is not explicitly mentioned in the Singapore Statement. Anticipation, according to Ruggiu [45], refers to planned action that makes provision for or precludes other action. It ‘seems to have become the key word in coping with the challenges of research and innovation which invest in new and emerging technologies' (p. 53) says Ruggiu, and, as such, carries much weight in contemporary RR(I) frameworks. This is reflected in how anticipation carries weight across different research disciplines (despite possibly being interpreted and applied in slightly different ways).

In the literature reviewed, anticipation seemed to be an important consideration for responsible research in technoscientific innovation and information technology, due to both the uncertainty and potential scale of their innovations' impacts on society. Moor, who has written much on the topic of technology and ethics, highlights the importance of anticipation in technology as developments get a foothold in society, stating that ‘as technological revolutions increase their social impact, ethical problems increase’ [46, p. 117]. In technoscientific innovation, argue Owen *et al.* [47], anticipation can improve foresight in risk assessment by encouraging researchers to deeply consider the potential effects of research which involves human–machine interfacing, and making them more aware of the social and ethical implications.

In a related area, but with different concerns altogether, van der Burg [48] considers anticipation in the context of a particular innovation: the use of imaging technology for breast cancer screening. Using research resources responsibly and balancing arguments for their purchase against other competing (financial) needs in research and other contexts can be complex. Van der Burg highlights that anticipating and reflecting on this complexity is part of RCR practice. She cautions that in cases where certain technology is sensitive and specific enough to correctly detect cancer, the use of those technologies may grow beyond the financial capacities for smaller practices, and to consider what that might mean for funding for other important medical equipment (and, by extension, other patients needing machine-delivered treatment).

Brey's [49] paper on anticipation in information technology explores uncertainty, pointing out the challenge that exists for fields (such as those in nanotechnology) where little is known about how a technology will affect society in the future. Brey articulates the nuances of some of the challenges, arguing that while it is undesirable for ethicists to get lost in speculation over the uncertainties of future possible ethical dilemmas, it is also undesirable for them to stay silent about emerging technologies simply because of those uncertainties.

Harm minimization is a dimension of RCR closely related to anticipation and is frequently discussed in the context of a harm–benefit balance. Along with informed decision-making and clarity, Roco *et al.* [50] discuss how anticipation can be exercised in practice to inform the achievement of a good balance between reducing uncertainty that will hamper nanotechnological advances and ensuring the responsible development and use of technologies, especially if they may raise concerns for the environment, health or safety. McLeod [34] ties in anticipation with the harm–benefit balance in relation to animal research. She discusses how the harm–benefit analysis must seriously engage with anticipated harms to animals and points out that, historically, too much weight has been given to the ‘promissory benefits to health and biomedicine’ (p. 10) and not enough to the anticipation of harm to animal health and well-being. With respect to human rights in research governance, others have argued the opposite, that policies tend to err on the side of protecting against individual exploitation rather than ‘promoting social and economic development that situates genomics research and applications as a public good that enhances human capabilities and economic productivity’ ([51, p. 899]; see also [52]).

It should be noted that, although anticipation and harm minimization featured prominently in literature from the RRI tradition, little mention is made of the concept in RCR frameworks or literature on research integrity. This is unsurprising, as anticipation largely focuses on the potential impacts of research outputs on society, a consideration central to RRI but outside the scope of research integrity. However, these two traditions may benefit from cross-pollination, as building anticipation into the process of conducting research could be valuable to the aims of promoting the trustworthiness of research.

### 4.9. Capacity building

Responsibility 13 of the Singapore Statement briefly mentions that ‘… research institutions should create and sustain environments that encourage integrity through education…’; however, the RCR dimension of capacity building is clearly much more prominent in the literature. Importantly, with one exception, the articles that emphasize capacity building as part of RCR were almost exclusively published within the last five years of the 30-year span of this literature (i.e. 2017–2022). This indicates that this is an emerging property of RCR and, as such, is not an expected part of the Singapore Statement.

Capacity building involves developing the skills and resources of a person or other entity and facilitating their use and further development in a particular context. It encompasses the facilitation of training, education, mentorship and support (financial and otherwise), and can be seen as occupying a spectrum ranging from institutional responsibility to individual responsibility. It aims both to improve the integrity of research itself, as well as facilitate the relevance and responsiveness of research to those involved in the research process and to broader society. Regarding RCR, which we have already discussed is relational (that is, ‘responsibility’ implies another party that someone is responsible *to*), capacity building takes a slightly shifted perspective on RCR compared with many of the dimensions already discussed here. To illustrate, rather than capacity building being only the responsibility of an individual researcher to society, it is also (perhaps more so) the responsibility of an institute to their employees, or of a research group to the indigenous population with which they are conducting participatory research. That responsibility surrounding capacity building should in most cases be a bidirectional transaction, highlights the importance of a reciprocal openness to learning and development.

Many of the articles that discuss capacity building focus on indigenous research and the responsibilities of institutions. Kombe and colleagues [53] discuss the promotion of research integrity in Africa and explore problems of academic misconduct in research in parts of Africa. Kombe *et al*. discuss capacity building as part of a proposed strategy to improve research conduct. They talk about developing syllabi for African university students (both under- and post-graduates) that foreground ethics and responsible research practice, and they recommend that ethics training certificates be made a requirement for anyone applying for approval to conduct research studies. Courses must be developed, they argue, to train already-qualified scientists to conduct responsible research, and training must be kept up to date. In a related point, they underscore the need to provide guidelines and standards to facilitate the implementation of such education and training.

Kombe and colleagues [53, p. 9] comment on the important role of mentorship in the capacity-building process: ‘Best practices should be sought and identified, and a formalized mentorship program should be developed to guide senior researchers on how best to mentor undergraduate and postgraduate students, postdoctoral fellows or other research staff more effectively’. Capacity building, they argue, is a key approach to address scientific misconduct and to promote responsible research. Although these articles centre the voices of the developing world in the RCR discourse, their arguments are broadly applicable.

Several articles in the corpus explore the issue of capacity building in the context of working with particular communities of people. Consider the research of Wagner and colleagues [54], for instance, who present an article on fostering RCR on ancient DNA (or aDNA). They argue that researchers who work with ancient human remains (including their DNA) must prioritize working with the community due to the societal implications of such research. ‘[Ancient] DNA holds great potential for helping us understand the human experience in new and important ways', they argue (p. 183). They recommend that researchers work to build trust and respect in relationships with the community, and that they train students and other members of the community in research techniques. They point out that while many opportunities exist for such activities, the best way to engage with the community will be subject to their needs, wishes and ‘bandwidth’. They also suggest capacity building for the research team themselves: for researchers who have not been trained in community-based research approaches and ethics to seek training and build up their own competencies. In comparison with some of the other articles in the corpus, these authors place the responsibility of capacity building on researchers, rather than institutions.

Matson and her colleagues, who describe their research on *Manoomin*, approach capacity building from a different angle. They discuss the importance of inviting Native students, who already ‘already walk with a foot in each world’ [30, p. 113] to collaborate and learn as part of the research project, as they will develop their own skills while also offering to the research their experience in integrating worldviews that sometimes might seem at odds. These diverse students, they argue, serve as teachers on how to work together across wide-ranging academic and cultural contexts. They will take the learned research skills with them as they ‘become leaders of the next generation of collaborative researchers, resource managers, and policy-makers’ (p. 113). The researchers must be sure to give their support to these students as many of them will need to challenge traditional research methodology as well as break socio-economic barriers. As with Wagner and colleagues [54], Matson *et al*. consider capacity building an individual responsibility.

Most researchers would need to learn to become teachers and mentors to support capacity building in their research with indigenous groups and aDNA, as the two aforementioned articles discuss. Institutions, however, have a natural role as capacity-builders, as they tend to be equipped with resources as well as a motivation to upskill their employees. De Peuter and Conix [27] explore this element of RCR in their article on cultivating a culture of research integrity for institutions. Policies, procedures, codes of conduct and guidelines, in and of themselves, are insufficient in stimulating RCR practice, they argue. According to De Peuter and Conix [27], these reifications of culture ‘. . . should be embedded in a comprehensive institutional policy that stretches as far as managerial support and ethical leadership’ (p. 3). Fostering RCR relies on many aspects of support, training and facilitation, and is a responsibility on both the institutional and individual level.

For instance, facilities should provide specific and high-level training and support in methodology and statistics to improve research design, methodology and analysis [36]. According to both Mejlgaard *et al*. [55] and De Peuter and Conix [27], there is a great need for better supporting and training supervisors in their own training and mentorship of students. Institutes must provide digital facilities to support transparency and safe and ethical data management and storage [55,56] and sufficient and dedicated human resources [36]. These suggestions help RCR to avoid the pitfall mentioned by Valkenburg *et al*. [5], of operationalizing interventions for systemic problems at an individual level (e.g. of teaching).

## 5. Discussion

The qualitative analysis of the 75 articles in the final sample produced an interesting selection of dominant themes. While these elements are not central (or in some cases even present) in the Singapore Statement, or other similar frameworks we discussed earlier, they are salient in this literature body, which indicates that they may be candidate dimensions to consider for subsequently developed frameworks on RCR (such as the one our overarching project aims to generate). Recall that the sample is negatively skewed across the publication year variable, with half of articles being published in the last 6 years of the year range (which was 29 years). That this corpus yielded an ‘updated’ view of RCR is perhaps not surprising.

In distilling these themes from the literature in our scoping review, we faced the challenge of finding the right levels of depth and specificity. We aimed to identify dimensions of RCR that are broad enough to have meaning across many, if not most or all, instances of research, yet granular enough to pick apart the concept of RCR into distinct aspects that are meaningful to consider on their own.

*Integrity*, one of the Singapore Statement's four principles, emerged as a dominant theme in our analysis and is central to both early *and* more contemporary conceptualizations of RCR. It can be considered, as argued earlier, a meta-responsibility, in that it permeates many elements of RCR practice and theory and is broadly considered to be a foundational element of scientific practice and culture. Our analysis found that integrity was ubiquitously mentioned, but definitions of the term could vary dramatically in scope, from the relatively simple meaning of ‘trustworthiness’ to broad definitions that become almost synonymous with RCR.

For our purposes, we find it useful to treat integrity as a reasonably closely bounded concept tied to engendering trust and confidence in research, but to remember that it is a term used flexibly across the literature and practical contexts.

*Accountability* is one of the Singapore Statement's core principles which featured heavily in the corpus as a high-level theme. Within the Singapore Statement, the notion of internal accountability (i.e. accountability driven by individual actions, or internal motivations) is indirectly embedded in some of the responsibilities, for instance, in sharing findings and record-keeping. However, it is not directly mentioned, nor heavily emphasized, and a focus on external accountability (i.e. accountability driven by external motivators such as guidelines and policies) is not present at all. Accountability (especially of the external kind) is directly central to many of the articles in this corpus, however, especially for research that focuses on indigenous communities, the environment and nanotechnology, among other fields. It may be considered in future frameworks on RCR as a responsibility, rather than a core principle, as it can be directly, concretely applied in many contexts.

*Reflexivity* is a meta-responsibility which, although not present in the Singapore Statement, may find a place in frameworks established in the future. It enhances several other dimensions of RCR, including accountability, anticipation and transparency, as the corpus shows. Mentions of reflexivity mainly arose in the literature regarding the relationship of research to broader society, but greater reflexivity in practice of RCR would also increase integrity of research itself.

*Transparency* is a third meta-responsibility to consider as part of an updated framework. Probably driven by the recent push for open science and reform, interest in transparency appears to be at the core of many contemporary codes of conduct and guides for RCR.

*Anticipation* and *harm minimization* are two related dimensions that were represented strongly in this review. These were often mentioned in the context of emerging and risky fields of research and should play an important role in research which explores human-machine interactions (such as in health technology) in contemporary guides and ethics. Growing concerns about ethics in the context of big data and advancing technology highlights the need for greater anticipation and harm minimization in research conduct. As such, this dimension would be relevant to future RCR frameworks.

*Capacity building*, which encompasses training, mentorship and support, was another ‘new’ RCR dimension not mentioned in the Singapore Statement, but that may merit inclusion in future frameworks, possibly as a meta-responsibility given that it impacts upon and works within other dimensions. In the literature, it was discussed in connection with disciplines that engage with protected or vulnerable communities, and as part of the role of institutions.

Together, these themes cover a variety of aspects of RCR that are already prominent in the related literatures of research integrity and RRI. Our scoping review therefore synthesizes aspects of largely separate scholarly discourses, and aims to lay the foundation for a larger project that will use interviews and a Delphi process to create a mapping of how RCR is understood to apply across diverse disciplines and locales (as mentioned at the end of this paper's Introduction). Such a broadly applicable mapping will need to navigate the complexities encountered by any cross-disciplinary framework, of applying simultaneously to highly disparate research paradigms. Some frameworks have previously taken the approach of keeping guidelines general enough to apply across (almost) all disciplines. However, our aims necessitate we take a slightly different tack, as follows.

Our mapping is intended to be descriptive, yet in capturing the shared understanding of RCR, a concept inherently tinged with the prescriptive notion that we ought to conduct research responsibly, it is perhaps natural that we also aim for it to be practical enough to usefully guide practice. To meet the aim of describing differences across disciplines, our mapping will necessarily contain some dimensions which are only relevant to certain research paradigms (e.g. interpretive versus positivist paradigms), and will be marked as such. On the other hand, to avoid becoming completely unwieldy, cross-disciplinary mappings such as ours cannot fully cover the specificities of individual disciplines. Therefore, to be used as a guide, our mapping will need an extra layer of adaptation at the point of use to be interpreted for a user's particular context. This could be done by individuals using the mapping, but alternatively, groups or organizations particular to a certain discipline could create an extension to our mapping, to standardize best practice for their constituents.

A similar challenge arises when considering how our mapping will apply across not just disciplinary, but national and cultural boundaries. Previous literature acknowledges the many tensions and paradoxes of attempting to apply frameworks of RRI across countries (e.g. [57,58]). Although it was not within the remit of our scoping review, nor the larger project, to attempt to comprehensively do this for RCR, we can at least acknowledge that an extra level of interpretation and adaptation will be necessary to align our mapping of RCR within other cultural contexts, so it may give rise to local meaning-making and practice development.

## 6. Limitations

In addition to the challenges mentioned above in the larger aim of mapping RCR, there are certain limitations to the approach we have taken in this article. Firstly, it must be emphasized that this is a scoping review, not a systematic review intended to provide an exhaustive overview of all relevant literature. Accordingly, we searched for literature in a limited subset of all possible databases. Although this is accepted practice, we certainly will have missed some of the relevant literature. Similarly, there is undoubtedly more literature that is relevant to our topic but not captured by the search terms we used (although we argue that this will have been somewhat mitigated by our use of the snowball search strategy).

Our selection of sources was limited to English language. Although abstracts were screened by two authors, who discussed instances of unsure relevance, the full text screening was done by only one author. This renders the final decisions regarding source inclusion subject to a single viewpoint. Similarly, analysis was done by a single coder. This latter point is not necessarily a limitation, as that reflects a valid methodological choice in qualitative research, but it may be useful to reiterate for reflexivity's sake that our resultant themes will necessarily reflect this researcher's viewpoint.^10^ Lastly, we wish to acknowledge our choice to limit the scope of analysis: we have kept the themes relatively high level and have chosen not to delve into all possible themes that could have been discussed. Although this aids in concision, it necessarily leaves out many nuances that could have been included in a more comprehensive review. See appendix B, however, for an overview of all code groups that were generated during the analysis process.

## 7. Conclusion

What we know about RCR has been developed largely in disciplinary silos. This limits the spread and ultimate uptake of important RCR concepts that can be embedded in different research cultures (e.g. that are found in institutions, or disciplines). Not only that, but the current frameworks on RCR that inform codes of conduct for institutions and organizations, and guide individuals' research practices, are relatively dated and specific to quantitative sciences. We aim to go beyond disciplinary walls (in this study as well as the overarching project it belongs within) to get an understanding of common language and concepts, and to broaden and diversify how we see RCR, such that it can become an integral part of academic research culture. We also use the literature to explore RCR dimensions that are underemphasized or missed in existing frameworks. We intend for this scoping review to form the basis for further research on RCR dimensions, as well as the development of updated and more diverse frameworks for RCR conduct in research.

## Data Availability

Collected data, analysis code and materials are available at: https://proto.io/8ntex/.

## Footnotes

1 See https://www.wcrif.org/guidance/singapore-statement.

2 We would like to point out that the scoping review method was originally developed with clinical studies in mind, and that much of the guidance material available is most applicable to medical journal article types (see https://jbi-global-wiki.refined.site/space/MANUAL/4687833/11.1+Introduction+to+Scoping+reviews for an explanation of the background of the scoping review), and not the kinds of articles our scoping review was targeting. As such our scoping review does not perfectly adhere to these guidelines in every small detail. However, we do report our work in as much detail and with as much transparency as possible, in keeping with the spirit of the current guidelines of scoping reviews, where there is a: ‘need for this type of knowledge synthesis to be rigorously conducted, transparent and trustworthy’.

3 We reasoned that the literature body would be self-limiting, as discussions of this topic only began to gain traction in the research sphere in the early 1990s with the ethical, legal and social aspects of emerging science and technology (ELSA) framework.

4 By ‘endorsed’, we mean documents from institutions such as universities or other bodies, such as national organizations like the UK Reproducibility Network, that are not part of the scientific literature *per se*, but which are produced and disseminated by those institutions, and whose content is therefore ratified by those institutions. We would also like to clarify that we did not consider articles from researchers affiliated with an institution as being endorsed by that institution unless the work had been peer-reviewed.

5 Please note that although this is an exclusion criterion, we only realized it after the fact and had not planned it in advance as an exclusionary criterion as part of our protocol (see link here: https://osf.io/vy684).

6 While there is a lot to be said about this from an RCR perspective, we will refrain from doing so here.

7 The listed guidance documents can be found at https://www.wcrif.org/guidance/hong-kong-principles, https://www.wcrif.org/guidance/amsterdam-agenda and https://www.wcrif.org/guidance/montreal-statement, respectively.

8 Note that similar findings would probably apply to other new technology, but that will have been missed due to our deliberate exclusion of studies focusing heavily on innovation alone.

9 See https://www.go-fair.org/fair-principles/ for a guide to the FAIR principles of responsible data stewardship.

10 Though, S.M.F. herself practised reflexivity throughout the data processing phase, to mitigate the undue impact of her own biases as much as is possible.

## Appendix A. List of 75 articles included in the final sample for this scoping review

See references [3,5,14,19–21,23,24,26–30,33,35,36,41–43,48,50,51,53–55,59–121]

## Appendix B. Code groups and themes for the analysis of the materials in this scoping review

Of 202 codes, a total of 20 code groups were made. Of these, six groups were turned into themes as they were most substantial in terms of the codes within them, and in terms of their cohesiveness as a theme.

**Table:**

code group name	number of codes
Integrity	20
Transparency/openness	20
Capacity-building	16
Reflexivity	13
Accountability	12
Anticipation	12
Care	9
Misconduct	9
Culture (research)	8
Ethics	8
Reporting (standards)	8
Fairness/credit	7
Consent	6
Diversity and inclusivity	6
Honesty	6
COI	5
Dissemination	5
Leadership	4
Freedom	3
Sustainability	3
